# Abnormal cation exchange in insulin-resistant patients with essential hypertension

**Published:** 2008-04

**Authors:** DR Taylor, JR Wing, MI Sonnekus, FJ Milne, M Toman

**Affiliations:** Department of Internal Medicine, University of the Witwatersrand and Johannesburg Hospital, Johannesburg; Department of Internal Medicine, University of the Witwatersrand and Johannesburg Hospital, Johannesburg; Department of Internal Medicine, University of the Witwatersrand and Johannesburg Hospital, Johannesburg; Department of Internal Medicine, University of the Witwatersrand and Johannesburg Hospital, Johannesburg; Department of Pathology, University of the Witwatersrand, Johannesburg

## Abstract

**Objectives:**

To identify important factors that may contribute to abnormal glucose tolerance in elderly patients with treated hypertension with primary reference to changes in the following parameters: calculated insulin resistance, endogenous insulin processing and secretion; platelet cation concentration and membrane ATPase activity.

**Design:**

Thirty-nine patients receiving antihypertensive therapy (including low-dose thiazide treatment) were compared to 13 normotensive, normoglycaemic control subjects. Total platelet cation concentration and membrane ATPase activity were measured and, following a 75-g oral glucose test, serum insulin, proinsulin and 31-32 des-proinsulin responses were measured in prospectively defined hypertensive patients with normal glucose tolerance (NG), impaired glucose tolerance (IGT) and diabetes mellitus (DM).

**Results:**

Of the total patient cohort, seven patients manifested newly diagnosed DM, 18 had IGT and 14 NG. Among the three groups, no difference in duration of drug use (thiazides and beta-blockers) was noted; BMI and waist-to-hip ratio increased progressively from NG to IGT to overt DM. Compared to NG patients, serum insulin responses were significantly greater in the IGT (all time points) and DM (two-hour measurements) subjects. Proinsulin and 31-32 des-proinsulin serum responses were likewise significantly higher in the IGT and DM groups. The derived measure of insulin resistance in the hypertensive patients showed a significant increase in the progression from NG to IGT and DM. Mean total platelet potassium concentration was reduced in the DM compared to the IGT and the control groups, while platelet sodium, calcium and magnesium concentrations showed no significant differences. Platelet membrane magnesium ATPase activity was significantly higher in the normotensive control versus the hypertensive group. Sodium, potassium and calcium ATPase activity showed no significant differences among the subgroups.

**Conclusion:**

Our findings support the strong link between essential hypertension, insulin resistance/hyperinsulinaemia and regional adiposity. Beta-cell dysfunction (hypersecretion and abnormal insulin processing) is manifest in the progression from normality to overt diabetes. The use of antihypertensive therapy (low-dose thiazides and cardioselective beta-blockers) possibly added diabetogenic effect(s). The reduction in platelet total potassium concentration paralleled the diabetic state while a reduced membrane magnesium ATPase activity correlated with the hypertensive state.

## Summary

Patients with essential hypertension often manifest abnormal glucose tolerance, including impaired glucose tolerance (IGT) and diabetes mellitus (DM), and a number of defined relationships between hypertension and abnormal glucose regulation have been documented. Firstly, as a component of the metabolic syndrome, essential hypertension is independently associated with increased insulin resistance akin to a reduction in the measured insulin-mediated glucose disposal rate evident in patients with type 2 diabetes.^1^,^2^ Secondly, the frequent association of insulin resistance and hyperinsulinaemia in patients with hypertension suggests that these metabolic changes may influence blood pressure control by several mechanisms, including enhanced sodium retention, sympathetic activation and also endothelial dysfunction.^3^,^4^ Thirdly, it has been proposed that a common mechanism is able to link abnormal blood pressure control, insulin resistance/hyperinsulinaemia and ion transport. 5,6 Lastly, it is also possible that antihypertension drugs, including the use of thiazide therapy combined with cardio-selective beta-blockers may contribute to abnormal glucose tolerance in hypertensive subjects.^7^

We have previously reported a high incidence of both IGT and DM in elderly patients with treated hypertension.^8^ The purpose of this present study was to identify some of the important clinical factors that may contribute to the abnormal glucose tolerance evident in hypertensive patients. We were specifically interested in documenting the measured changes in insulin resistance, endogenous beta-cell processing/secretion of insulin and platelet cation concentration and membrane ATPase activity in a cohort of thiazide-treated hypertensive patients with prospectively defined normal glucose tolerance, impaired glucose tolerance and hitherto undiagnosed and untreated type 2 diabetes mellitus.

## Patients and methods

Thirty-nine Caucasian patients (mean age 65.8 ± 2.7) with treated, well-controlled essential hypertension were studied. All had received antihypertensive therapy for a minimum of five years, which included low-dose thiazide therapy (hydrochlorothiazide 12.5 mg or 25 mg daily). A control group of 13 healthy normotensive subjects (mean age 56.2 ± 1.6) with normal glucose tolerance, matched for age, weight and waist−hip ratio (WHR) was also included in the study in order to compare total platelet cation concentrations and platelet ATPase activity. The means of the control group were compared with the means of each of the three groups of test subjects.

The following anthropomorphic measurements were recorded: weight (kg), body mass index (kg/m^2^), waist−hip ratio and waist alone (cm). Blood pressure was recorded in the sitting position with a standard, calibrated mercury sphygmomanometer and the mean of five separate measurements was obtained for each patient. After an overnight fast, the patients were challenged with a 75-g oral glucose tolerance test. Venous blood was withdrawn in the fasting state and hourly for two hours, where appropriate.

The following laboratory measurements were undertaken: serum glucose by a standard glucose oxidase method (Synchon CX3 analyser, Beckmann Instruments, Galway, Ireland); serum insulin concentration by radio-immunoassay (Medginex Diagnostics, SA, Fleuris, Belgium); intact proinsulin and 31-32 des-proinsulin concentration by an in-house, specific and sensitive two-site, immunoradiometric assay based on the method of Sobey, *et al*;^9^ serum potassium and lipids by standard reagent methods (Boehringer Mannheim, Mannheim, Germany); serum creatinine by the Jaffe method (Boehringer Mannheim, Mannheim, Germany); total platelet cation content was determined by previously described methods;^10^ sodium and potassium concentration was assessed by flame photometry (Model 543, Instrumentation Laboratories, Inc, Lexington, Mass, USA); total platelet calcium and magnesium by atomic absorption spectrophotometry (Varian Techtron AA 175 series with a Mg or Ca hollow cathode lamp); platelet isolation and membrane preparation were performed and ATPase activities for magnesium, calcium, sodium and potassium were assayed by established methods.^11^ The magnitude of insulin resistance was calculated using the homeostasis model assessment (HOMA) parameters.^12^

Unless otherwise specified, the results are expressed as the mean ± SEM. Statistical analyses included the following: analysis of variance for multiple comparisons; the Student’s *t*-test and Spearman’s correlation coefficient for paired samples; and Chi-squared test to assess significance in trend. Significance was taken at the 5% level. The study was undertaken with patient informed consent and after review and approval by the Committee for Research on Human Subjects, University of the Witwatersrand.

## Results

Based on the two-hour post-glucose challenge, as can be seen in Table 1, seven patients were found to have newly diagnosed diabetes mellitus with glucose measurements greater than 11.1 mmol/l. Eighteen patients manifested impaired glucose tolerance values between 7.8 and 11 mmol/l, and 14 subjects were confirmed to have normal glucose tolerance (NG) levels less than 7.7 mmol/l (fasting plasma glucose ≤ 5.6 mmol/l). These three subgroups of thiazide-treated hypertensive patients were compared to a control group of normotensive subjects (*n* = 13) with normal glucose tolerance (fasting plasma glucose ≤ 5.6 mmol/l).

**Table 1.** Demographic Data

	*NG*	*IGT*	*DM*
Total patient number	14	18	7
Men	4	5	1
Women	10	13	6
Age (years)	67.1 ± 2.8	68.2 ± 1.8	62.1 ± 3.6
Weight (kg)	71.6 ± 3.9	75.9 ± 4.2	76.8 ± 10.0
BMI (kg/m^2^)	26.8 ± 1.3	29.1 ± 1.7	28.5 ± 2.6
Waist−hip ratio	0.83 ± 0.02*	0.87 ± 0.02	0.92 ± 0.03*
SBP (mmHg)	151 ± 4.3	155 ± 3.9	145 ± 5.4
DBP (mmHg)	80 ± 1.8	84 ± 3.0	80 ± 2.0
Diuretic use (months)	67.9 ± 3.7	64.7 ± 6.5	66.6 ± 13.6
β-blocker use (months)	59.8 ± 8.9	70.1 ± 7.8	63.1 ± 11.3
Family history of diabetes (%)	5 (36)	5 (28)	5 (71)

*p < 0.004.

Within the hypertensive subgroups of patients (Table 1) there were no differences in either the mean total duration of thiazide therapy (DM = 66.6 ± 13.6 months; IGT = 64.7 ± 6.5 months; NG = 67.9 ± 3.7 months) or in the mean duration of β-blocker therapy (DM = 63.1 ± 11.3 months; IGT = 70.1 ± 7.8 months; NG = 59.8 ± 8.9 months). A family history of diabetes involving a first-degree relative was documented with the following frequency: DM = 71%; IGT = 28%; NG = 36%, without any statistical significance being evident. BMI and waist-to-hip ratio showed a progressive increase from NG to IGT and DM, but significance was only established in the greater waist-to-hip ratio between the DM and NG subgroups (DM = 0.92 ± 0.03; NG = 0.83 ± 0.02; *p* < 0.004).

A positive correlation was established between the following variables: weight with fasting glucose (*r* = 0.39, *p* < 0.05) and fasting insulin values (*r* = 0.43, *p* = 0.02); BMI with fasting glucose (*r* = 0.39, *p* < 0.05) and fasting insulin values (*r* = 0.52, *p* < 0.01); waist−hip ratio with fasting glucose levels (*r* = 0.61, *p* < 0.01); waist circumference with fasting glucose (*r* = 0.60, *p* < 0.01) and fasting insulin values (*r* = 0.66, *p* < 0.01) (data not shown).

**Fig. 1.**
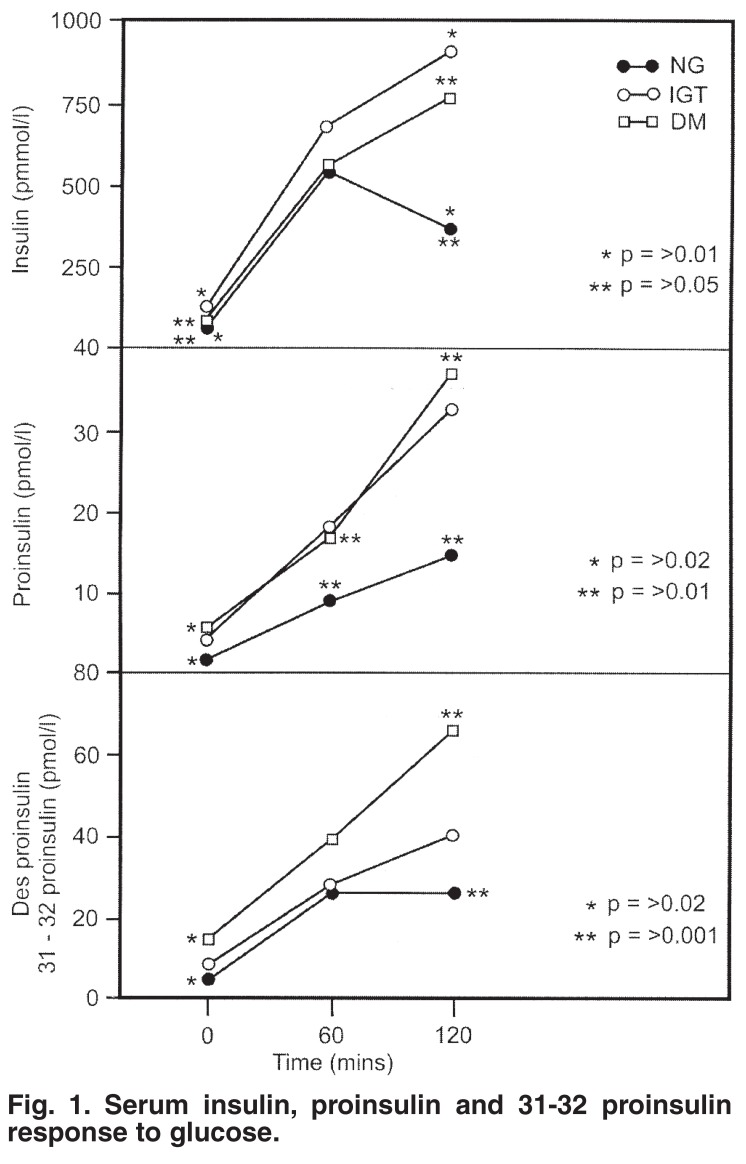
Serum insulin, proinsulin and 31-32 proinsulin response to glucose.

The serum insulin, proinsulin and 31-32 des-proinsulin measurements in response to the glucose challenge are shown in Fig. 1. In the top panel, the NG patients showed a typical time-related insulin response from zero to one and two hours, respectively, with the peak response occurring at one hour. In comparison to the NG patients, the IGT group exhibited a mean insulin response that was both increased and delayed such that ongoing hyperinsulinaemia was evident after the two-hour time point.

Significance between these two groups was established at zero and two hours, respectively, (NG vs IGT: 0 hr = 59.2 ± 4.4 and 121.6 ± 16.0 pmol/l, *p* < 0.01; NG vs IGT: 2 hr = 372.6 ± 68.0 and 932.7 ± 15.9 pmol/l, *p* < 0.01). The DM patients manifested dynamic insulin responses that were intermediate, being greater in magnitude and delayed in time at two hours in comparison to the NG patients. These changes were lower than those present in the IGT group and significance was established at the following time-points: NG vs DM: 0 hr = 59.2 ± 4.4 and 115.2 ± 36 pmol/l; *p* = 0.05; NG vs DM: 2 hr = 372.6 ± 68.0 and 764.7 ± 112 pmol/l; *p* = 0.05.

The mean serum proinsulin response (Fig. 1, middle panel) showed a progressive increase in both the fasting and post-stimulated state in the progression from NG to IGT and DM. Significant differences were evident at all time points, zero, one and two hours, respectively, between the NG and DM groups. A similar trend was present in the responses of the 31-32 split proinsulin measurements (lower panel) and significance was established between the NG and DM groups at two time points, zero and two hours.

The homeostasis model assessment (HOMA): based on the measured fasting insulin and glucose concentrations, a progressive increase in the calculated degree of insulin resistance was evident in the change from NG (2.27 ± 0.61 μU/ml × mmol/l) to IGT (3.92 ± 0.52 μU/ml × mmol/l) and DM (6.79 ± 1.2 μU/ml × mmol/l). Significance was established between the DM, IGT and NG groups (*p* < 0.001) as well as the DM and IGT groups (*p* < 0.01) (Table 2).

**Table 2.** Calculation Of Insulin Resistance Homeostasis Model Assessment (HOMA) Formulation

*Group*	*Number*	*Mean (μU/ml × mmol/l)*	*SEM*
Normal (NG)	13	2.27*	0.61
Impaired (IGT)	15	3.92*	0.52
Diabetic (DM)	7	6.79*	1.25

*p < 0.001.

Table 3 shows that the total platelet sodium, calcium and magnesium concentrations were similar in all the subgroups of hypertensive patients (NG, IGT and DM) and also in the normotensive controls with normal glucose tolerance (CG). In contrast, the mean total platelet potassium concentration in the hypertensive DM group (2.28 ± 0.05 μmol/l × 10^8^ cells) was significantly reduced in comparison to the hypertensive IGT (2.69 ± 0.03 μmol/l × 10^8^ cells; *p* = 0.02), hypertensive NG (2.65 ± 0.4 μmol/l × 10^8^ cells; *p* = 0.02) and normotensive control groups (2.63 ± .02 μmol/l × 108 cells; *p* < 0.01). Of note, this finding was evident in the presence of similar serum potassium concentrations in all subgroups.

**Table 3.** Total Intracellular Platelet Cation Concentrations In Normotensive Subjects With Normal Glucose Tolerance (CG) And Hypertensive Patients With Normal Glucose Tolerance (NG), Impaired Glucose Tolerance (IGT) And Diabetes Mellitus (DM)

*Cation (μmol/l × 10^8^ cells)*	*CG (n = 13)*	*NG (n = 14)*	*IGT (n = 18)*	*DM (n = 7)*
Sodium	1.0 ± 0.09	0.8 ± 0.08	0.9 ± 0.09	0.9 ± 0.15
Potassium	2.63 ± 0.02*	2.65 ± 0.04^#^	2.69 ± 0.03^#^	2.28 ± 0.05^#^
Calcium	0.65 ± 0.06	0.59 ± 0.09	0.45 ± 0.05	0.43 ± 0.05
Magnesium	0.92 ± 0.05	0.99 ± 0.12	0.92 ± 098	1.04 ± 0.17

*p = 0.02; ^#^*p* = 0.01.

Platelet ATPase activity (Table 4) showed no measured differences in sodium−potassium ATPase and calcium ATPase activity within and between the four subgroups: hypertensive NG, hypertensive IGT, hypertensive DM and normotensive CG. Platelet magnesium ATPase activity, however, showed an increased activity in the control group (non-hypertensive, normoglycaemic), which was weakly significant when compared against two of the three hypertensive groups (NG: *p* = 0.02 and IGT: = *p* 0.001 but not the DM group). Comparison between the control group and the combined hypertensive groups (NG + IGT + DM) confirmed a decrease in magnesium ATPase activity in the hypertensive group (70.64 ± 4.4 nmol Pi/mg protein/min) versus the normotensive controls 52.86 ± 3.6 nmol Pi/mg protein/min (*p* = 0.01).

**Table 4.** Platelet Atpase Activity In Normotensive Subjects With Normal Glucose Tolerance (CG) And Hypertensive Patients With Normal Glucose Tolerance (NG), Impaired Glucose Tolerance (IGT) And Diabetes Mellitus (DM)

*ATPase (nmol Pi/mg protein/min)*	*CG*	*NG*	*IGT*	*DM*
Na-K	7.33 ± 0.99	10.15 ± 1.50	10.29 ± 1.73	9.38 ± 2.46
Calcium	5.9 ± 0.87	5.23 ± 0.39	7.18 ± 0.96	8.7 ± 1.32
Magnesium	70.64 ± 4.4^#^	51.8 ± 6.2*	47.28 ± 4.1^#^	66.04 ± 8.7*

*p = 0.02; ^#^*p* = 0.001.

## Discussion

As with previously reported studies, we have documented a high incidence of abnormal glucose tolerance in our cohort of elderly treated patients with otherwise reasonably well-controlled essential hypertension.^8^ The overall incidence of abnormal glucose tolerance in our subjects was 64%, with the incidence of IGT being 46% and that of diabetes mellitus 18%. The changes in the WHO 1998 recommendation in the diagnostic value of fasting plasma glucose levels greater than 7.0 mmol/l made very little difference to this group of patients as only two subjects had fasting plasma glucose levels greater than 7.0 mmol/l. In this regard, it has been estimated that up to 50% of patients with type 2 diabetes remain undiagnosed and many of these may be included among subjects who fall into categories of the metabolic syndrome, which include, in particular, patients with ischaemic heart disease and hypertension. In addition, as many as a third of patients with primary hypertension will manifest with overt diabetes mellitus in time, and our incidence of diabetes is in keeping with these reported ranges.^13^-^18^

Within our patient groups, this high incidence of IGT and DM may be explained by two major factors, namely, increased age and overweight, and particularly, a centripetal weight distribution. The mean age of our cohort was 65.8 years and the background prevalence of type 2 diabetes is known to be high in many elderly populations. Based on the calculated BMI, our three groups of hypertensive patients were not overtly obese but were overweight. Nonetheless, even in this setting of modest overweight, a significant difference in the waist-to-hip ratio was evident between the NG and DM patients, attesting to the known importance of regional adiposity in the genesis of insulin resistance and diabetes.^19^,^20^ We were able to document positive and significant correlations between indices of adiposity, mean fasting glucose and insulin resistance (mean fasting insulin) in our hypertensive patients. We were therefore able to associate primary hypertension with a high incidence of the metabolic co-morbidities of glucose intolerance, diabetes and increased regional adiposity.

Although the presence of a positive family history of diabetes mellitus showed a progressive increase in prevalence from the NG to DM subjects, we were unable to assign statistical value(s) of significance to this trend. This was attributable to our relatively small patient numbers, but our findings do not negate the importance of a genetic/constitutional risk in the development of diabetes in subjects with hypertension. In this regard, it has been well established that normotensive, non-diabetic, first-degree relatives of hypertensive subjects demonstrate insulin resistance at a young age.^14^,^15^

We were also unable to document any adverse metabolic/diabetogenic effect(s) attributable to antihypertensive therapy (especially that of thiazide therapy) that may have contributed to the high incidence of IGT/diabetes in our patients.^9^,^21^-^23^ As all the patients were uniformly treated with thiazides, we assessed the total duration of drug exposure and found no significant differences in this parameter of drug ‘exposure’ between the three subgroups of patients. Thiazide therapy prescribed in low doses therefore seemingly had little or no impact on promoting glucose intolerance in these elderly and otherwise, ‘at-risk’ patients with hypertension. This is in contrast to other studies, 24-26 which suggest that the combination of thiazide (given in low daily doses) and β-blocker therapy (mainly atenolol) had an increased diabetogenic effect in comparison to angiotensin-converting enzyme (ACE) inhibitors, angiotensin II receptor blockers (ARBs) and calcium channel blockers (CCBs).

It has been documented that up to 50% of patients with essential hypertension will manifest insulin resistance/hyperinsulinaemia.^16^ In this setting it also seems likely that the incidence of hypertension is significantly greater in these ‘at-risk’ subjects who are insulin resistant than those who are insulin sensitive.^27^ Based on the plasma insulin responses to an oral glucose challenge and also on the HOMA-derived level of insulin resistance, we were able to identify subsets of hypertensive patients who were either insulin sensitive with normal glucose tolerance (35%), or insulin resistant with IGT or DM (65%), with the majority of the latter group manifesting pre-diabetes rather than overt diabetes. Serum insulin responses to an oral glucose challenge were therefore significantly different in magnitude and duration in the three groups of hypertensive patients with varying degrees of glucose tolerance/intolerance.

In comparison to the patients with normal glucose tolerance, the patients who manifested abnormal glucose tolerance showed an insulin response both increased and delayed in the IGT patients and reduced and delayed in the DM patients. The observed trend in the hypersecretion of endogenous insulin was paralleled by an increase in the HOMA calculation of insulin resistance which increased significantly, two-fold and three-fold in the progression from NG to IGT and DM, respectively.

The pathogenesis of type 2 diabetes is ascribed to both insulin resistance and pancreatic beta-cell dysfunction. This abnormality in insulin secretion is initially manifested as increased/exaggerated secretion as a compensatory response to insulin resistance (stages of IGT and pre-IGT), but which subsequently manifests a progressive decline in insulin secretion as beta-cell failure emerges and hyperglycaemia supervenes (DM). These linked stages of hyperinsulinaemia followed by glucose intolerance that occurred in our metabolically ‘at-risk’ patients with hypertension are therefore similar to the pathogenetic abnormalities evident in most patients who develop type 2 diabetes.

Apart from changes in insulin secretion, a noted manifestation of pancreatic dysfunction that has been identified in the pre-diabetic and diabetic state includes the hypersecretion of insulin precursors, specifically pro-insulin and 31-32 split proinsulin. It has also been demonstrated that these changes in the secretion of serum insulin precursor peptides occur incrementally as glucose intolerance deteriorates. We were also able to demonstrate that in hypertensive patients who manifest with varying degrees of glucose intolerance, a progressive and significant increase in the stimulated serum proinsulin and 31-32 split proinsulin concentrations was evident in the progression from NG to IGT and DM. Therefore, as beta-cell decompensation occurs in insulin-resistant hypertensive patients, a manifestation of this ‘β-cell stress’ becomes evident in the concomitant and parallel hypersecretion of insulin precursor peptides.

In our patient cohort, platelet cation activity showed two significant correlations: diabetes compared to non-diabetes was associated with a reduction in total platelet potassium concentrations, while hypertension compared to non-hypertension was associated with reduced platelet membrane magnesium ATPase activity. The pathogenetic relevance of these measured changes are uncertain and our findings need to be interpreted in the context of our small patient cohort and the potential role of antihypertensive therapy and dysglycaemia in modulating cation concentration.

No difference(s) in serum potassium concentration was evident between our patient subgroups, but of note, serum magnesium measurements were not undertaken and our findings may reflect relative magnesium deficiency between the control and treated hypertensive groups. Nonetheless, the regulatory role of intracellular magnesium in a number of cellular ion channels and pumps that modulate peripheral vascular tone has been well documented. In all of these instances, low intra-cellular magnesium levels or activity would potentiate calcium-dependent vasoconstriction^28^ and underlie a dual dysregulation of the sodium−magnesium exchanger that is present in patients with essential hypertension.^29^

Previous data in a similar small group of South Africans found increased, albeit not significant platelet membrane ATPase activity, especially magnesium ATPase activity in hypertensive versus normotensive subjects.^11^ Our own data seemingly fit the hypothesis that insulin resistance/hyperinsulinaemia is able to modulate transmembrane cation flux in susceptible patients and thereby influence vascular tone, and also provide a unifying mechanism that explains the link between hypertension and glucose intolerance.^30^,^31^

## Conclusion

Our findings support the strong link between hypertension, insulin resistance/hyperinsulinaemia and regional adiposity. It is possible that the use of thiazide and β-blocker therapy may have had an added adverse impact on glucose tolerance/intolerance. The metabolic sequel to this relationship is manifest in the very high incidence of glucose intolerance and diabetes in elderly patients with essential hypertension. The calculated magnitude of the level of insulin resistance in this group of patients increased progressively as deterioration in glucose tolerance supervened. The indices of pancreatic beta-cell dysfunction paralleled a reduction in insulin sensitivity and were characterised by increased and prolonged endogenous insulin secretion and perturbed beta-cell processing manifest in the increase in stimulated serum insulin precursor peptide (proinsulin and 31-32 des-proinsulin) concentrations. The changes in intracellular cation activity reflected both the diabetic and hypertensive states, respectively, and conform to the unifying hypothesis that abnormal cation exchange, hypertension and insulin resistance co-exist.
